# Gastric adenosquamous carcinoma producing granulocyte-colony stimulating factor: a case of a rare malignancy

**DOI:** 10.1186/s40792-017-0338-7

**Published:** 2017-05-11

**Authors:** Kazuki Moro, Masayuki Nagahashi, Tetsuya Naito, Yu Nagai, Tomohiro Katada, Masahiro Minagawa, Jun Hasegawa, Tatsuo Tani, Naohiro Shimakage, Hiroyuki Usuda, Emmanuel Gabriel, Tsutomu Kawaguchi, Kazuaki Takabe, Toshifumi Wakai

**Affiliations:** 1 Division of Digestive and General Surgery, Niigata University Graduate School of Medical and Dental Sciences, 1-757 Asahimachi-dori, Chuo-ku, Niigata City, Niigata 951-8510 Japan; 2 Division of Digestive and General Surgery, Japanese Red Cross Nagaoka Hospital, Nagaoka City, Niigata 940-2085 Japan; 3 Division of Diagnostic Pathology, Japanese Red Cross Nagaoka Hospital, Nagaoka City, Niigata 940-2085 Japan; 4 Department of Surgical Oncology, Roswell Park Cancer Institute, Buffalo, NY 14263 USA; 5 Department of Surgery, University at Buffalo Jacobs School of Medicine and Biomedical Sciences, the State University of New York, Buffalo, NY 14203 USA

**Keywords:** Adenosquamous carcinoma, Gastric cancer, Granulocyte-colony stimulating factor

## Abstract

**Background:**

A gastric adenosquamous carcinoma (ASC) that produces granulocyte-colony stimulating factor (G-CSF) is an uncommon malignancy with a poor prognosis. Due to the rarity of this lesion, a standard treatment for the disease has not been established.

**Case presentation:**

We describe a case of a 66-year-old male with a G-CSF-producing gastric ASC who presented with severe anemia and leukocytosis. A radical resection was performed, followed by a course of adjuvant chemotherapy. Histopathologic examination revealed that the tumor consisted of areas of both squamous cell carcinoma and adenocarcinoma. Immunohistochemical staining with an anti-G-CSF antibody was also positive. He was started on adjuvant capecitabine and oxaliplatin (CapeOX) 6 weeks after surgery. The patient stopped treatment after 3 months due to his own preference. Eight months following surgery, the patient was found to have diffuse lymph node, liver, and peritoneal metastases.

**Conclusions:**

G-CSF-producing gastric ASC is a rare and aggressive tumor. Because patients are usually diagnosed at an advanced stage, multidisciplinary evaluation and innovative treatments are needed. The rarity of this disease, with its aggressive features, poses a significant challenge in its treatment. In this brief case report, we summarize the management and outcomes of G-CSF-producing gastric ASC.

## Background

Gastric cancer is the fourth most prevalent cancer in the world and the second most common cause of cancer-related deaths [1]. Gastric adenosquamous carcinoma (ASC) has characteristics of both adenocarcinoma and squamous cell carcinoma, with more than a quarter of the tumor typically composed of the squamous cell carcinoma component. It is an uncommon disease, representing less than 1% of all gastric adenocarcinomas [2]. It has a poor prognosis [3], with the 3-year overall survival rate reported to be 15.4% [2].

Expression of granulocyte-colony stimulating factor (G-CSF) and the G-CSF receptor (G-CSFR) by gastric cancers rarely occurs. G-CSF is a pro-inflammatory cytokine that stimulates myeloid stem cell maturation, proliferation, and migration into the circulation [4]. Despite being a known growth factor, the impact of G-CSF on gastric cancer has not been clearly established. However, G-CSF production is associated with a poor prognosis because G-CSF promotes malignant proliferation and invasion. It has been reported that the median overall survival is only 5 months (range, 2–38 months) [5].

G-CSF-producing gastric ASC has two poor prognostic factors, namely, the squamous cell component of the tumor and the G-CSF production. Although the incidence of G-CSF-producing gastric tumors is approximately 0.5% of all pure gastric cancers, it has a very poor prognosis [6]. Interestingly, in contrast to this low rate of ASC in pure gastric adenocarcinoma, the rate of ASC in G-CSF-producing gastric cancer is significantly higher and has been reported to be up to 14.3% [7]. In this report, we describe a rare case of G-CSF-producing gastric ASC.

## Case presentation

A 66-year-old male with a history of type 2 diabetes, chronic hepatitis, and 2 years of anemia despite iron supplementation, presented with a generalized and worsening fatigue over a 1-month period, which necessitated an emergency department visit. The patient also experienced unintentional weight loss of 10 kg over 3 months. He had a history of smoking and drinking for 30 years. He had a benign physical exam. On laboratory evaluation, he had severe anemia (hemoglobin 4.7 g/dl) and leukocytosis (white blood cell 12900/μL, with 68.4% neutrophils). Serum C-reactive protein (CRP) was elevated to 1.61 mg/dl (normal range 0.01–0.30).

**Fig. 1:**
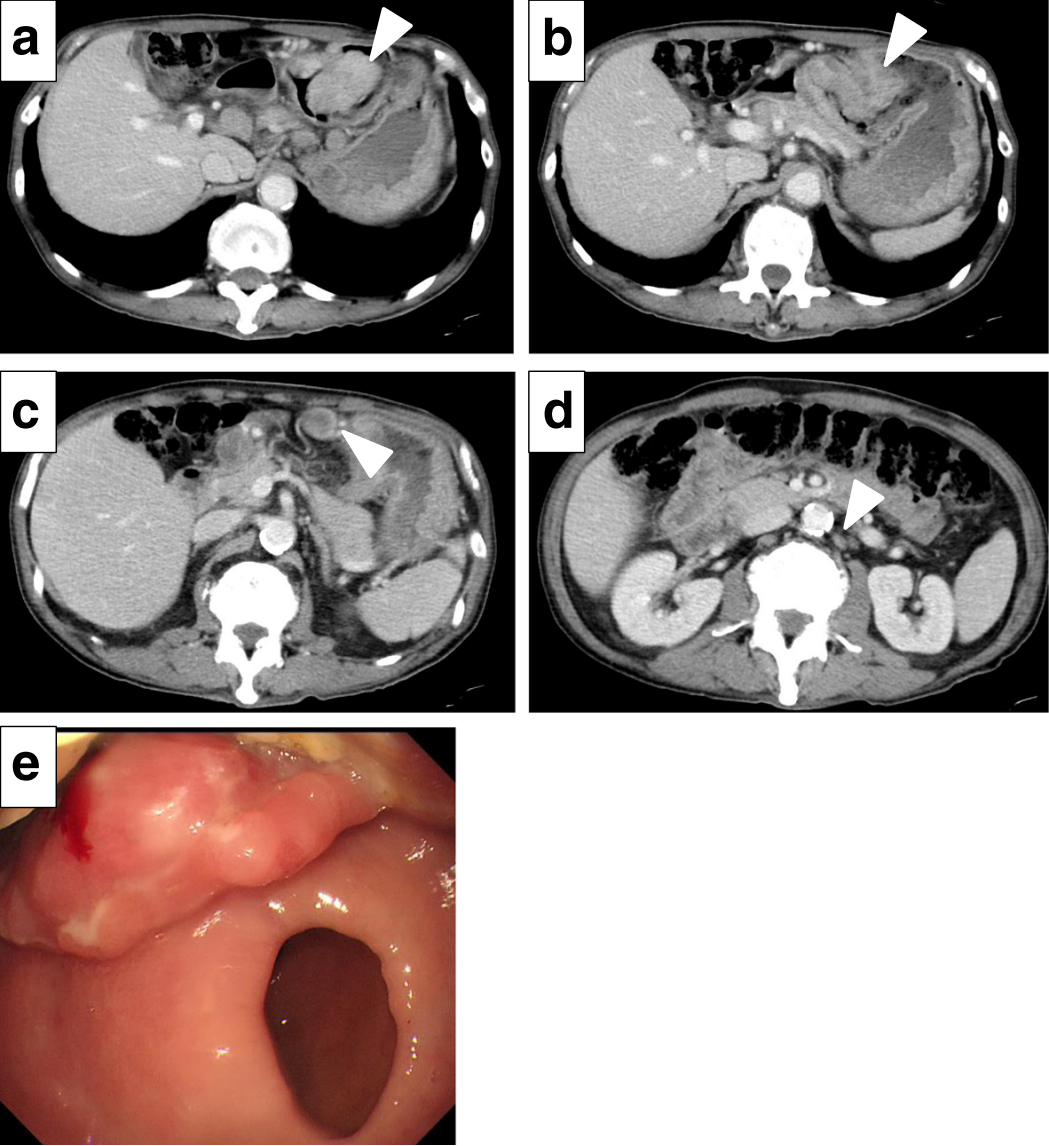
Examination images of the gastric cancer before the operation. **a** Abdominal computed tomography revealing an enhancing mass in the gastric antrum (*white arrow head*). **b** A more distal section of the gastric tumor with evidence of pyloristenosis (*white arrow head*). **c** Peri-gastric lymph node enlargement (*white arrow head*). **d** Enlarged para-aortic lymph nodes (*white arrow head*). **e** Esophagogastroduodenoscopy revealing type 2 gastric cancer approaching the pylorus

A computed tomography (CT) scan of the abdomen and pelvis demonstrated irregular thickening of the antrum wall with surrounding para-aortic lymph node enlargement (Fig. 1a–d). There was no evidence of distal metastasis. Esophagogastroduodenoscopy demonstrated a type 2 tumor in the gastric antrum with ulceration (Fig. 1e). The endoscopic needle biopsy showed a poorly differentiated adenocarcinoma.

**Fig. 2:**
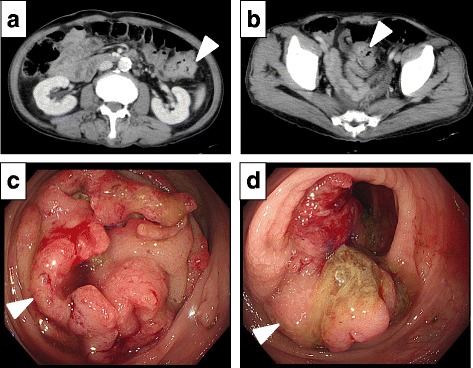
Examination images of the colon cancers before the operation. **a** Abdominal computed tomography revealing an enhancing mass in the sigmoid colon (*white arrow head*). **b** A second enhancing mass was identified in the descending colon (*white arrow head*). **c** Colonoscopy revealing sigmoid colon cancer (*white arrow head*). **d** Synchronous descending colon cancer (*white arrow head*)

Coincidentally, the CT scan also demonstrated irregular thickening of the colon wall, in two separate areas within the descending and sigmoid colon with surrounding lymph node enlargement (Fig. 2a, b). A colonoscopy showed synchronous descending colon and sigmoid colon cancers (Fig. 2c, d). Thus, the patient presented with synchronous gastric cancer and two colon cancer foci.

We planned to resect both gastric cancer and the two colon cancer foci simultaneously. Because some para-aortic lymph nodes were only slightly enlarged, we suspected that these nodes represented a reactive, inflammatory process as opposed to obvious metastases from either the gastric or colon cancers. Although a PET-CT scan was considered, it was not completed since we did not have access to a PET-CT scanner in our institution at that time. The patient underwent an open subtotal gastrectomy accompanied by a D2 lymphadenectomy (including superior mesenteric vein lymph node resection and middle colic artery lymph node resection), a gastrojejunal Roux-en-Y anastomosis, and a left colectomy accompanied by extended lymphadenectomy (D3)—including para-aortic node lymph node sampling—to address the slight preclinical suspicion of lymph node involvement from either the gastric or colonic primary.

**Fig. 3:**
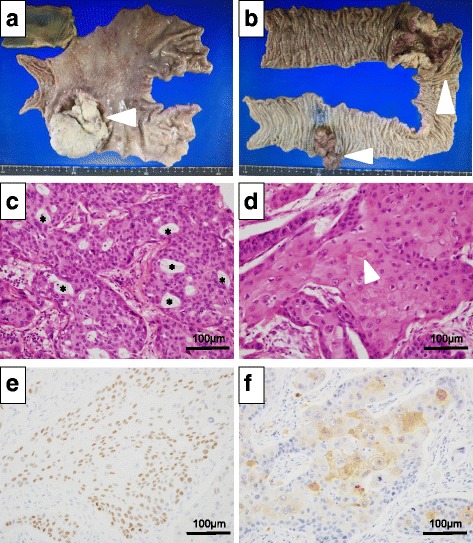
Histopathology of gastric adenosquamous carcinoma. **a** Gross specimen of gastric cancer. **b** Gross specimen of sigmoid colon cancer and descending colon cancer. **c** Glandular structure (*asterisk*) showing adenocarcinoma components (hematoxylin and eosin staining, ×200 magnification). **d** Single-cell keratinization showing squamous cell carcinoma components (hematoxylin and eosin staining, 200× magnification) (*white arrow head*). **e** Immunohistochemical staining for p40 (**e**, ×200 magnification). **f** Staining for G-CSF (**f**, ×200 magnification) is positive in the squamous cell carcinoma components

An 8.0 × 8.0 × 6.0 cm type 2 tumor was identified in the antrum (Fig. 3a). A 6.5 × 5.5 cm and 6.5 × 3.0 cm type 2 tumors were identified in the sigmoid colon and descending colon respectively (Fig. 3b). Histologically, the gastric tumor was confirmed to be an ASC, composed of both adenocarcinoma and squamous cell. The former showed glandular structures (Fig. 3c) and intercellular bridges, while the latter showed single-cell keratinization (Fig. 3d). Both components were intermingled. Immunohistochemically, the tumor was also positive for p40, a squamous differentiation marker (Fig. 3e) consistent with the diagnosis of an ASC. Because the rate of ASC in G-CSF-producing gastric cancer is high (14.3%) [7], we additionally decided to stain for G-CSF. Immunohistochemical analysis revealed positive focal staining with an anti-G-CSF antibody (Fig. 3f). The tumor was negative for anti-HER2 antibody staining. Histopathological examination of the superior mesenteric and middle colic artery nodes showed metastatic disease consistent with the ASC from the gastric tumor, but not from the colon cancer. Thus, the patient had a diagnosis of pathologic stage IV (T4a N3b M1) G-CSF-producing gastric ASC. The descending colon cancer consisted of a moderately differentiated tubular adenocarcinoma, pathologic stage II (T3 N0 M0). The sigmoid colon cancer consisted of a well-differentiated tubular adenocarcinoma, pathologic stage I (T1b N0 M0).

**Fig. 4:**
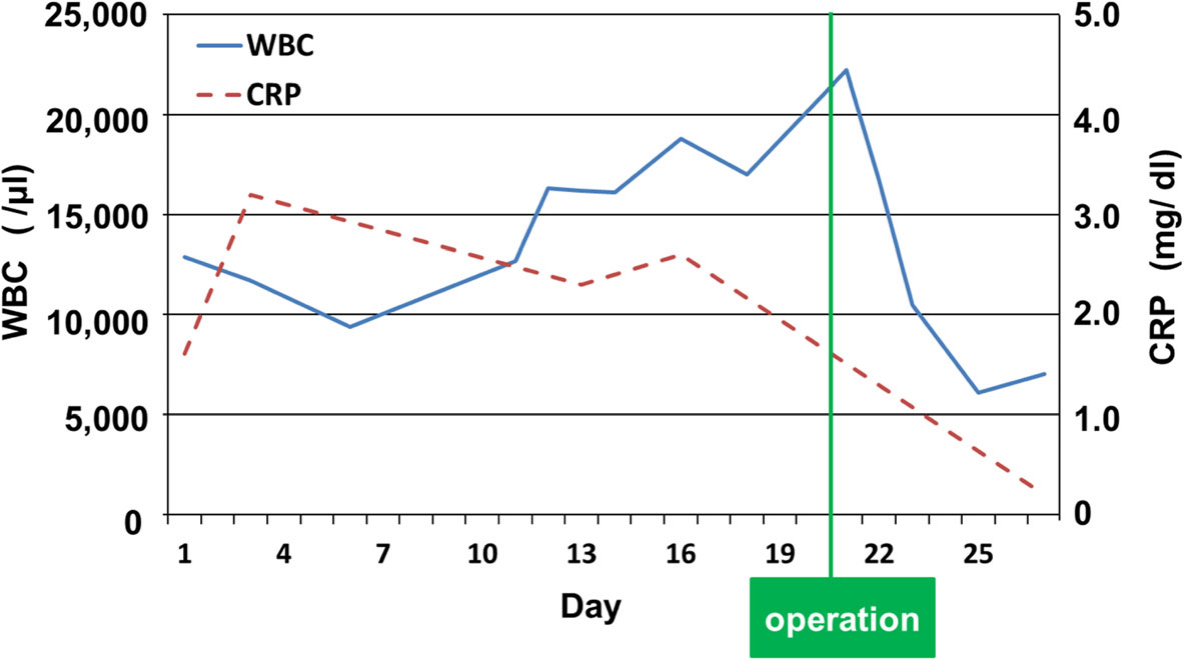
The trends of white blood cell (WBC) count and C-reactive protein (CRP) levels. Both values are normalized postoperatively

Although preoperatively the patient had a leukocytosis, this normalized after surgery (Fig.4). Similarly, his CRP also normalized following surgery. The patient was discharged without any complication on postoperative day 17. He was started on adjuvant capecitabine and oxaliplatin (CapeOX) 6 weeks after surgery. The patient stopped treatment after 3 months at his own preference. He did not report any drug toxicities. Eight months following surgery, the patient’s cancer recurred and he was found to have diffuse body lymph node, liver, and peritoneal metastases on his most recent follow-up imaging.

### Discussion

Gastric ASC consists of a mixture of adenocarcinoma and squamous cell carcinoma components [2]. In rare cases, these may also produce G-CSF. G-CSF, a potent hematopoietic cytokine, regulates the proliferation and differentiation of granulocytic progenitor cells and functionally activates mature neutrophils. G-CSF affects non-hematopoietic tumor cells by binding to G-CSF-specific receptors on their surfaces. In a cancer setting, G-CSF functions as an autocrine growth factor on malignant cells through activation of the G-CSF receptor [8–10]. Because of the squamous component and G-CSF production, the prognosis for G-CSF-producing gastric ASC is worse than for pure adenocarcinoma.

These aspects of G-CSF-producing ASC, together with the very low incidence of this disease, pose challenges for treatment. There are few reports on G-CSF-producing ASC and no established standard treatment for this very rare disease. Takahashi et al. reported that chemotherapy (S-1 and CDDP) treated the adenocarcinoma component but not the squamous component and therefore suggested that sensitivity to chemotherapy may differ between adenocarcinoma and squamous cell carcinoma [11].

The most common sites of metastases in gastric ASC are the liver and intra-abdominal lymph nodes [12]. In Japanese cases of G-CSF-producing gastric cancers, 42% of patients had a liver metastasis and 28% of patients had a direct invasion of the adjacent organ [11]. In this case report, the G-CSF-producing gastric ASC was ultimately found at an advanced stage, similar to those reported in other publications. Although the staging CT scan identified suspicious para-aortic lymph nodes, the patient had two synchronous colon cancers. Therefore, it was highly challenging to determine if these lymph nodes had metastatic disease and whether it originated from the gastric or colon cancers. Furthermore, because the patient presented with a severe, symptomatic anemia, a top priority was to control bleeding. Consequently, even with a slight but real possibility of metastatic disease, we decided to proceed with symptomatic resection and sample the suspicious para-aortic nodes during the surgery. In general, the prognosis with curative resection in advanced gastric cancer is superior to the prognosis with noncurative resection [13]. Considering that the frequency of peritoneal metastasis is less in G-CSF-producing gastric ASC than in pure gastric adenocarcinoma [11], the prognosis is expected to improve with aggressive resection as long as all disease can be removed. In this case, however, there were lymph node metastases found in the para-aortic lymph nodes, resulting in an unlikely curative resection. Indeed, this was the case, as the patient’s cancer recurred within 8 months following surgery with widespread metastases.

Due to the complicated management of this disease, multidisciplinary therapy that includes surgery and chemotherapy is often considered. There are few reports of chemotherapy in G-CSF-producing gastric ASC, and the outcomes of G-CSF-producing gastric ASC with surgery and adjuvant chemotherapy are suboptimal. Table 1 summarizes the case reports of G-CSF-producing gastric ASC, where most patients exhibit a very rapid deteriorating disease course within a few postoperative months. In these reports including our case, radiation therapy was never performed. Considering that squamous cell carcinoma is generally sensitive to radiation therapy, there may be benefit of radiation therapy specifically for the squamous component of gastric ASC. Currently, however, no study has been able to demonstrate the effectiveness of radiation even in ASC of the esophagogastric junction most likely due to the rarity of the disease. Therefore, at this time, we luck sufficient evidence to recommend radiation therapy for gastric ASC.

**Table 1:** Summary of the reported cases of granulocyte-colony stimulating factor-producing gastric adenosquamous cell carcinoma

Case	Author	Year	Age	Sex	WBC (/μl)	G-CSF (pg/ml)	Stage	Chemotheraphy	Prognosis
1	Koyama	1993	51	M	36, 600	128	ND	ND	ND
2	Okada	2001	56	M	21, 000	72	IV	5-FU, Cisplatin	ND
3	Nasu	2004	62	M	14, 100	64	IB	ND	31 months alive
4	Endo	2005	55	M	35, 000	105	IIIA	S-1, CPT-11, PTX	23 months dead
5	Sato	2007	67	M	19, 090	91	II	S-1	5 months alive
6	Ikemoto	2007	67	M	21, 500	391	IV	S-1	ND
7	Saito	2012	65	M	21, 970	89	IV	Doxetacel	3 months dead
8	Our case	2016	66	M	12, 900	ND	IV	Capecitabine, Oxaliplatin	8 months alive

*CPT-11* irinotecan, *5-FU* 5-fluorouracil, *G-CSF* granulocyte-colony stimulating factor, *M* male, *ND* not described, *PTX* paclitaxel, *S-1* tegafur/gimeracil/oteracil, *WBC* white blood cell

Saito et al. reported treatment of their G-CSF-producing gastric ASC case using a combination of chemotherapy with docetaxel, cisplatin, and S-1 (DCS). Interestingly, an early antitumor effect was observed that correlated to the normalization of the WBC count. However, the tumor relapsed after the cessation of chemotherapy. In our case, the WBC count also normalized after surgery. Thus, the WBC count may serve as a potential biomarker for G-CSF-producing gastric ASC, though the number of cases is low to make this a strong claim.

## Conclusions

G-CSF-producing gastric ASC is a rare and aggressive tumor. Because these patients are usually diagnosed at an advanced stage, multidisciplinary evaluation and innovative treatment approaches are needed to improve their long-term outcomes.

## Abbreviations

ASC: Adenosquamous carcinoma
CDDP: Cisplatin
CRP: C-reactive protein
CT: Computed tomography
DCS: Docetaxel, cisplatin, and S-1
G-CSF: Granulocyte-colony stimulating factor
G-CSFR: G-CSF receptor
PET: 18-Fluorodeoxyglucose positron emission tomography
S-1: Tegafur/gimeracil/oteracil
